# A Bayesian network meta‐analysis of the efficacy of targeted therapies and chemotherapy for treatment of triple‐negative breast cancer

**DOI:** 10.1002/cam4.1892

**Published:** 2018-12-07

**Authors:** Huihui Chen, Wei Lu, Yixin Zhang, Xuan Zhu, Jiaojiao Zhou, Yiding Chen

**Affiliations:** ^1^ Department of Surgical Oncology, The Second Affiliated Hospital Zhejiang University School of Medicine Hangzhou Zhejiang China; ^2^ Department of Thyroid and Breast Surgery Yinzhou People Hospital Ningbo Zhejiang China; ^3^ The Key Laboratory of Cancer Prevention and Intervention China National Ministry of Education Hangzhou Zhejiang China

**Keywords:** network meta‐analysis, randomized controlled trials, targeted therapies, triple‐negative breast cancer

## Abstract

Triple‐negative breast cancer (TNBC) is a heterogeneous disease with poorer prognosis than other subtypes, yet effective therapies are still not available. We aimed to compare the efficacy of various targeted therapies with chemotherapy (CT) in TNBC patients using a network meta‐analysis. A systematic literature search was performed in PubMed, EMBASE, and the Cochrane Library. A total of 27 randomized controlled trials (RCTs), involving 6924 TNBC patients, were included. Olaparib significantly improved PFS (0.43, 0.29‐0.64) and ORR (2.57, 1.31‐5.09) in comparison with CT. As for bevacizumab + CT, it showed a significant improvement of PFS (0.66, 0.55‐0.80) and ORR (2.15, 1.16‐4.05) compared with CT + placebo. It was also superior to CT alone in PFS (0.48, 0.35‐0.65) and pCR (1.30, 1.13‐1.49 for breast and axillary nodes and 1.26, 1.11‐1.44 for breast). Other targeted agents like iniparib, sorafenib, cetuximab, and ipatasertib combined with CT showed significant superiority in PFS compared with CT alone, and the HRs were 0.75 (0.62‐0.90), 0.44 (0.21‐0.91), 0.67 (0.47‐0.96), and 0.44 (0.24‐0.81), respectively, while some other agents such as sunitinib and cetuximab had the lowest SUCRA in OS, PFS, or ORR without any benefits. In conclusion, our results indicated that the addition of bevacizumab to CT was beneficial for TNBC patients, and olaparib had a great effect in PFS and ORR, especially for those with BRCA mutations. When combined with CT, targeted agents including iniparib, sorafenib, cetuximab, and ipatasertib may have better efficacies for treating TNBC.

## 1 INTRODUCTION

Breast cancer is the most common malignancy in women, with an estimated 1.67 million new cases diagnosed and 522 000 deaths worldwide in 2012.^1^, ^2^ Triple‐negative breast cancer (TNBC) represents 15% of breast cancers and is defined by the absence of estrogen receptor (ER), progesterone receptor (PR), and human epidermal growth factor receptor 2 (HER2).^3^ Notorious for its aggressiveness, TNBC is associated with a higher mortality than other subtypes.^4^ Due to its molecular characteristics, TNBC patients can not benefit from endocrine and anti‐HER2 therapies.^5^ Some studies revealed that many TNBC patients were highly sensitive to chemotherapy and had better neoadjuvant response rates compared with others,^6^, ^7^ suggesting chemotherapy may be an alternative therapeutic strategy. However, there is a high risk of relapse and disease progression after chemotherapy, and TNBC patients with residual lesions have significantly worse survival compared with non‐TNBC patients.^7^ Consequently, no standard therapy existed for TNBC patients and discovering effective targeted agents for this patient population has been a particularly urgent need.

In recent years, increased knowledge of the molecular alterations in TNBC has led to several promising clinical approaches, including inhibitors of poly ADP‐ribose polymerase (PARP), angiogenesis, epidermal growth factor receptor (EGFR), etc, which are currently being evaluated and may shed new light on treatment strategies.^5^, ^8^ Bevacizumab, a directed vascular endothelial growth factor A (VEGF‐A) antagonist, was chosen as a candidate treatment.^8^ Addition of bevacizumab to chemotherapy was reported to improve response rates and time to progression among TNBC patients.^9^, ^10^ Preclinical and clinical studies have shown that BRCA‐mutated tumors had increased responses rates to PARP‐inhibitor therapy.^11^ Even with a lack of a BRCA mutation, a substantial proportion of TNBC patients showed biological similarities with BRCA‐associated breast cancers,^5^, ^12^ making it possible that PARP inhibitors could be applied to TNBC patients. In the neoadjuvant I‐SPY2 trial,^13^ veliparib combined with carboplatin increased pCR rates from 26% to 51% in the TNBC group. Apart from all the above, some other molecular targeted drugs such as perbrolizumab (a monoclonal antibody targeting PD‐1), glembatumumab vedotin (an antibody‐drug conjugate [ADC] targeting transmembrane glycoprotein NMB [gpNMB]), are attracting researchers’ attention, as well.^5^, ^14^, ^15^

Two previous traditional meta‐analyses were performed to explore the issue of whether the targeted therapy combined with chemotherapy (CT) was superior to CT alone.^16^, ^17^ However, there were many limitations in using the pairwise meta‐analysis. First, among all available treatments, a lack of head‐to‐head trials makes direct comparisons of certain treatments impossible. Second, study endpoints and targeted therapies are various among different trials. Bayesian network meta‐analysis enables indirect comparison using a common comparator when a head‐to‐head trial is not available, and it produces estimation of the relative effectiveness and rank ordering among all interventions.^18^, ^19^ Thus, we performed a network meta‐analysis to compare different study endpoints of various targeted agents in the treatment of TNBC.

## 2 MATERIALS AND METHODS

### 2.1 Literature search strategy

This study was designed in accordance with the instructions of the preferred reporting items for systematic reviews and the meta‐analysis (PRISMA) extension statement incorporating network meta‐analyses of healthcare interventions.^20^ PubMed, EMBASE, and the Cochrane Collaboration Central Register of Controlled Clinical Trials were searched, and the search strategy was provided in the Supporting Information. Bibliographies of the included trials and related reviews were also manually scanned for additional references.

### 2.2 Selection criteria

We included only randomized controlled clinical trials (RCTs) assessing the efficacy of targeted therapies alone or in combination with chemotherapy for TNBC patients. Study end outcomes included overall survival (OS), progression‐free survival (PFS), disease‐free survival (DFS), objective response rate (ORR) and pathological complete response (pCR), and hazard ratios (HRs) or relative risks (RRs) with their corresponding 95% confidence intervals (CIs) when available or could be calculated. Single arm studies, non‐English literature, duplicated studies, and studies without sufficient outcome measures were excluded.

### 2.3 Data extraction and assessment for risk of bias

Two abstractors (Huihui Chen and Wei Lu) independently extracted data from the included studies. Disagreements were resolved by discussion with a third reviewer until consensus was reached. We collected information about the first author, year of publication, the number of patients in each arm, age, TNM stage, ECOG performance status, neoadjuvant chemotherapy or not, treatment regimens, study endpoints, and their relevant HRs or RRs. If HRs and 95% CIs were indirectly reported in the form of Kaplan‐Meier survival curves, we estimated them according to Guyot's methods.^21^ For different follow‐up durations in the same trial, only data with the longest duration were adopted. Methodological risk of bias of individual studies was assessed by using the Cochrane risk of bias method, including random sequence generation, allocation concealment, blinding of participants and personnel, blinding of outcome assessors, incomplete outcome data, selective reporting, and other types of bias,^22^ and each part was evaluated as low risk, unclear risk, and high risk of bias.

### 2.4 Data synthesis and analysis

In the first place, network plots showed direct comparisons between treatment arms and offered a brief description of their characteristics. The influence and contribution of each direct comparison to the whole network was then presented with contribution plots.^23^ In addition, we adopted comparison‐adjusted funnel plots to assess the potential small‐study effects in the network meta‐analysis.^23^

For Bayesian network meta‐analysis, we built the Bayesian framework based on the log‐hazard ratio scale and the posterior distribution of the treatment effect was estimated using Markov Chain Monte Carlo methods.^24^ Both fixed and random effects models were tested in the primary analysis, and the selection of these two models was based on deviance information criteria (DIC) and convergence of the model. DIC was used to quantify the fit of the fixed or random effects model,^19^ with differentials of 2 to 5 between the random effects model and fixed effects model being considered important.^25^ Convergence of the model was examined by using Brooks‐Gelman‐Rubin diagnostic methods, inspecting autocorrelation between iterations of the Markov chain and determining whether the error of effect size was less than 5% of the posterior standard deviation.^26^ All Bayesian network analyses were performed using two chains, and after discarding the results of a burn‐in period of 40 000 iterations, each chain had a sample of 200 000 iterations which were then thinned every 20th iteration to reduce autocorrelation. Vague priors, such as N(0, 10^6^) for the study‐specific baseline and treatment effect coefficients used to insure estimates of effect sizes and precision, were informed by within‐study differences between treatments rather than by differences in absolute response between the studies.^24^ The median and 95% credible intervals (CrIs) of the posterior distribution were recorded as estimates for effect sizes, and credible intervals could be interpreted as conventional CIs. The probability and the cumulative probability of rank for each treatment were calculated, and the surface under the cumulative ranking curve (SUCRA) was adopted to compare the efficacy of each treatment.^27^

To evaluate whether there was inconsistency between indirect and direct comparisons, we compared the estimated effect sizes from Bayesian network meta‐analysis with the corresponding pooled effect sizes from traditional pairwise meta‐analysis. The extent of heterogeneity across studies was tested using the *I* ^2^ test, and *I* ^2^ > 50% together with *P* < 0.05 indicated significant heterogeneity.^28^ We adopted a random effects model if significant heterogeneity existed; otherwise, we adopted a fixed effects model. For sensitivity analysis, we conducted network meta‐analysis based on the frequentist graph‐theoretical model.^29^, ^30^ Like the SUCRA value in Bayesian network meta‐analysis, the P‐score was used for ranking treatments in the frequentist network meta‐analysis without resampling.^31^

The Bayesian network meta‐analysis was performed using the Markov Chain Monte Carlo engine WinBUGS software (MRC Biostatistics Unit, Cambridge, UK), RevMan 5.0 software (The Cochrane Collaboration, Copenhagen, Denmark), Stata 12.0 (StatCorp, College Station, TX, USA), and R 3.2.1 (R development Core Team, Vienna, http://www.R-project.org) with the “netmeta” package (V.0.9‐2).^30^ The statistical significance level was set to *P* < 0.05 unless specified.

## 3 RESULTS

### 3.1 Study selection work flow and summary of characteristics of the included trials

**Figure 1:**
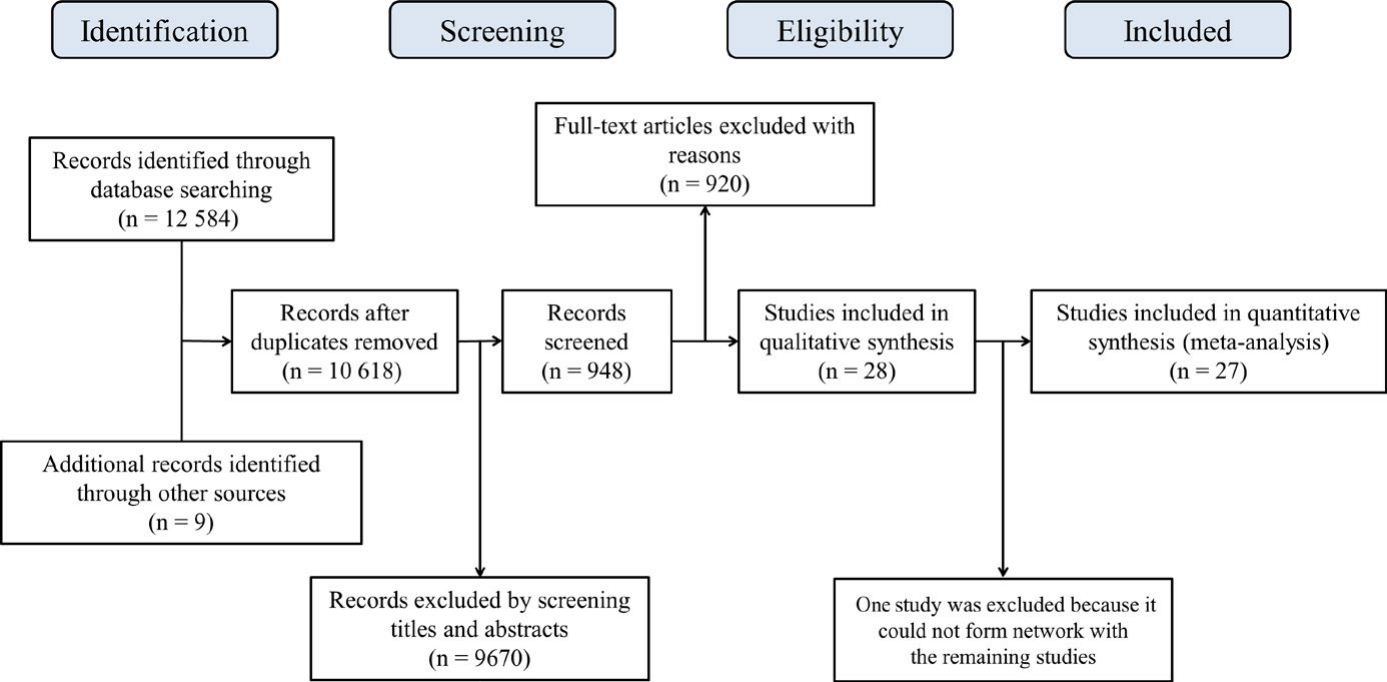
Flow diagram of literature search and study selection. A total of 27 randomized controlled trials that met the inclusion criteria were included in the network meta‐analysis

As shown in Figure 1, a total of 12 584 studies through electronic database searches and nine studies from relevant studies or reviews were identified. After removing duplicated studies and initial screening, we retrieved the full text of 948 potentially eligible articles for detailed assessment, and 920 studies were excluded according to the inclusion and exclusion criteria. The treatment arms of the screened 28 studies were recorded, yet Dieras’ study, which compared the efficacy between onartuzumab + bevacizumab + CT, bevacizumab + CT + placebo, and onartuzumab + CT + placebo for TNBC patients, was excluded because the treatments did not share common nodes with the others.^32^ Finally, 27 RCTs involving 6924 TNBC patients were included and the characteristics of the included trials are displayed in Table 1.^9^, ^10^, ^15^, ^33^, ^34^, ^35^, ^36^, ^37^, ^38^, ^39^, ^40^, ^41^, ^42^, ^43^, ^44^, ^45^, ^46^, ^47^, ^48^, ^49^, ^50^, ^51^, ^52^, ^53^, ^54^, ^55^, ^56^

**Table 1:** Characteristics of the included studies

Author‐year	Trial identifier	Median age (y)	TNM Stage	ECOG	Neoadjuvant chemotherapy	Number in arm 1	Number in arm 2	Arm 1	Arm 2	End points	HR for OS	HR for PFS	RR for ORR	RR for pCR breast and axillary nodes	RR for pCR breast
Baselga‐2012	2007‐000290‐32 (EudraCT)	NA	IIIB, IIIC, IV	0‐1	No	20	33	Sorafenib and capecitabine	Placebo and capecitabine	PFS	NA	0.60 (0.31‐1.14)	NA	NA	NA
Baselga‐2013	NCT00463788	Arm 1:53 Arm 2:52	IV	0‐2	No	115	58	Cetuximab and cisplatin	Cisplatin	OS, PFS, ORR	0.82 (0.56‐1.20)	0.67 (0.47‐0.97)	1.93 (0.83‐4.48)	NA	NA
Bell‐2016	NCT00528567	Arm 1:50 Arm 2:50	T1b‐T3	0‐2	No	1301	1290	Bevacizumab and standard chemotherapy	Standard chemotherapy	OS, DFS	0.93 (0.74‐1.17)	NA	NA	NA	NA
Bergh‐2012	NCT00393939	NA	Locally recurrent, IV	0‐2	No	58	69	Sunitinib and docetaxel	Docetaxel	PFS	NA	1.03 (0.65‐1.63)	NA	NA	NA
Brufsky‐2012	NCT00281697	Arm 1:55 Arm 2:49	IV	0‐1	No	112	47	Bevacizumab plus taxane or gemcitabine or capecitabine or vinorelbine	Placebo plus taxane or gemcitabine or capecitabine or vinorelbine	OS, PFS, ORR	0.62 (0.39‐1.01)	0.49 (0.33‐0.74)	2.14 (1.14‐4.02)	NA	NA
Carey‐2012	NCT00232505	Arm 1:52 Arm 2:49	IV	0‐2 and unknown	No	71	31	Cetuximab and carboplatin	Cetuximab	OS, PFS, ORR	0.93 (0.57‐1.50)	0.49 (0.23‐0.64)	2.62 (0.62‐11.02)	NA	NA
Curigliano‐2013	NCT00246571	Arm 1:52 Arm 2:52	Locally recurrent, IV	0‐1 and ≥2	No	113	104	Sunitinib	Capecitabine or vinorelbine or docetaxel or paclitaxel or gemcitabine	OS, PFS, ORR	1.16 (0.86‐1.56)	1.20 (0.89‐1.63)	0.39 (0.10‐1.49)	NA	NA
Finn‐2009	NCT00075270	NA	III, IV	0‐2 and unknown	No	71	60	Lapatinib and paclitaxel	Placebo and paclitaxel	PFS	NA	1.25 (0.85‐1.83)	NA	NA	NA
Forero‐2015	NCT01307891	Arm 1:51 Arm 2:50	IV	NA	No	39	21	Tigatuzumab and nab‐paclitaxel	Nab‐paclitaxel	PFS, ORR	NA	1.27 (0.76‐2.19)	0.74 (0.35‐1.55)	NA	NA
Gonzalez‐2014	NCT00499603	Arm 1:46 Arm 2:52	IIA‐IIIC	NA	Yes	23	27	Everolimus plus paclitaxel, 5‐fluorouracil, epirubicin, and cyclophosphamide	Paclitaxel, 5‐fluorouracil, epirubicin, and cyclophosphamide	ORR, pCR	NA	NA	0.76 (0.50‐1.16)	1.17 (0.48‐2.85)	NA
Gray‐2009	NCT00028990	NA	IV	NA	No	arm1+arm2: 232	arm1+arm2: 232	Bevacizumab and paclitaxel	Paclitaxel	PFS	NA	0.49 (0.34‐0.70)	NA	NA	NA
Han‐2018	NCT01506609	NA	Locally recurrent, IV	0‐2	No	40	42	Veliparib plus carboplatin and paclitaxel	Placebo plus carboplatin and paclitaxel	PFS	NA	0.82 (0.47‐1.40)	NA	NA	NA
Jovanovic‐2017	NCT00930930	Arm 1:52 Arm 2:52	II, III	NA	Yes	96	49	Everolimus plus cisplatin and paclitaxel	Placebo plus cisplatin and paclitaxel	DFS, ORR, pCR	NA	NA	0.89 (0.77‐1.04)	0.74 (0.50‐1.10)	NA
Kim‐2017	NCT02162719	Arm 1:54 Arm 2:53	Locally recurrent, IV	0‐1	No	62	62	Ipatasertib and paclitaxel	Placebo and paclitaxel	PFS,ORR	NA	0.60 (0.37‐0.98)	1.25 (0.78‐2.00)	NA	NA
Kummar‐2016	NCT01306032	54	IV	0‐1	No	21	18	Veliparib and cyclophosphamide	Cyclophosphamide	PFS, ORR	NA	0.57 (0.21‐0.81)	1.71 (0.17‐17.38)	NA	NA
Llombart‐2015	NCT01204125	NA	II‐IIIA	0‐1	Yes	94	47	Iniparib and paclitaxel	Paclitaxel	ORR, pCR	NA	NA	1.04 (0.78‐1.38)	0.85 (0.42‐1.71)	0.95 (0.48‐1.88)
Minckwitz‐2012	NCT00567554	Arm 1:49 Arm 2:48	T1c‐T4d	NA	Yes	323	340	Bevacizumab plus epirubicin, cyclophosphamide, and docetaxel	Epirubicin, cyclophosphamide, and docetaxel	pCR	NA	NA	NA	1.32 (1.08‐1.60)	1.28 (1.07‐1.54)
Nahleh‐2016	NCT00856492	NA	IIB‐IIIC	NA	Yes	32	35	Bevacizumab plus nab‐paclitaxel, doxorubicin, cyclophosphamide, and pegfilgrastim	Nab‐paclitaxel, doxorubicin, cyclophosphamide, and pegfilgrastim	OS, PFS, pCR	0.49 (0.19‐1.29)	0.46 (0.20‐1.05)	NA	2.08 (1.14‐3.78)	NA
O'Shaughnessy‐2011	NCT00540358	Arm 1:56 Arm 2:53	IV	0‐1 and unknown	No	61	62	Iniparib plus gemcitabine and carboplatin	Gemcitabine and carboplatin	OS, PFS, ORR	0.57 (0.36‐0.90)	0.59 (0.39‐0.90)	1.63 (1.06‐2.51)	NA	NA
O'Shaughnessy‐2014	NCT00938652	Arm 1:53 Arm 2:54	Locally recurrent, IV	0‐2	No	261	258	Iniparib plus gemcitabine and carboplatin	Gemcitabine and carboplatin	OS, PFS, ORR	0.85 (0.69‐1.04)	0.79 (0.65‐0.98)	1.12 (0.87‐1.43)	NA	NA
Pivot‐2011	NCT00333775	NA	IV	0‐1	No	60	43	Bevacizumab and docetaxel	Placebo and docetaxel	PFS	NA	0.68 (0.46‐1.00)	NA	NA	NA
Robert (Cape)‐2011^a^	NCT00262067	NA	Locally recurrent, IV	NA	No	87	50	Bevacizumab and capecitabine	Placebo and capecitabine	PFS	NA	0.72 (0.49‐1.06)	NA	NA	NA
Robert (Tax/Anthra)‐2011^a^	NCT00262067	NA	IV	NA	No	96	46	Bevacizumab and standard chemotherapy	Placebo and standard chemotherapy	PFS	NA	0.78 (0.53‐1.15)	NA	NA	NA
Robson‐2017	NCT02000622	NA	IV	0‐1	No	102	48	Olaparib	Standard therapy	PFS, ORR	NA	0.43 (0.29‐0.63)	2.58 (1.30‐5.11)	NA	NA
Sikov (No carbo)‐2015^b^	NCT00861705	NA	II, III	NA	Yes	105	107	Bevacizumab plus paclitaxel, doxorubicin, and cyclophosphamide	Paclitaxel, doxorubicin, and cyclophosphamide	pCR	NA	NA	NA	1.02 (0.74‐1.40)	1.20 (0.90‐1.61)
Sikov (With carbo)‐2015^b^	NCT00861705	NA	II, III	NA	Yes	110	111	Bevacizumab plus paclitaxel, doxorubicin, cyclophosphamide, and carboplatin	Paclitaxel, doxorubicin, cyclophosphamide, and carboplatin	pCR	NA	NA	NA	1.34 (1.05‐1.71)	1.27 (1.02‐1.57)
Tredan‐2015	NCT00633464	Arm 1:50 Arm 2:53	Locally recurrent, IV	NA	No	39	40	Cetuximab and ixabepilone	Ixabepilone	ORR	NA	NA	1.20 (0.64‐2.25)	NA	NA
Yardley‐2015	NCT01156753	NA	III, IV	0‐3	No	28	11	Glembatumumab vedotin	Standard chemotherapy	OS, PFS, ORR	0.65 (0.29‐1.45)	0.69 (0.32‐1.54)	4.55 (0.27‐76.05)	NA	NA
Yardley‐2016	NCT01427933	NA	III, IV	0‐1 and ≥2	No	21	22	Ramucirumab and eribulin	Eribulin	PFS	NA	0.66 (0.32‐1.35)	NA	NA	NA

HR for OS or PFS was presented as arm1 vs arm2, and HR < 1 indicated patients in arm1 achieved better OS or PFS than patients in arm2. RR for ORR or pCR was presented as arm1 vs arm2, and RR > 1 indicated patients in arm1 achieved more ORR or pCR than patients in arm2.

CI, confidence interval; CT, chemotherapy; NA, not available; ORR, objective response rate; OS, overall survival; pCR, pathological complete response; PFS, progression‐free survival.

a Study Robert (Cape)‐2011 and study Robert (Tax/Anthra)‐2011 were from the same trial, and their CT was different, as shown in the table above.

b Study Sikov (No carbo)‐2015 and study Sikov (With carbo)‐2015 were from the same trial, and their CT was different, as shown in the table above.

**Figure 2:**
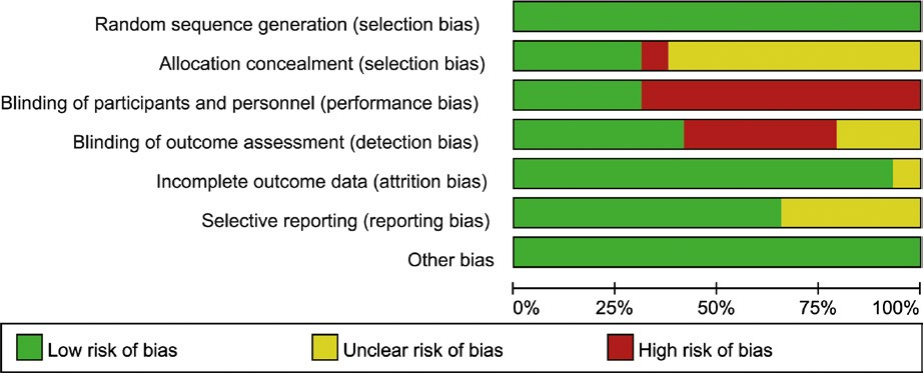
Methodological risk of bias assessment of the included studies in this network meta‐analysis. Methodological risk of bias contained the following part: random sequence generation (selection bias), allocation concealment (selection bias), blinding of participants and personnel (performance bias), blinding of outcome assessors (detection bias), incomplete outcome data (attrition bias), selective reporting (reporting bias), and other bias. Each part was evaluated as low risk of bias, unclear risk of bias, and high risk of bias. The length of the bar showed the percentage of total studies

For Robert's study, there were two intervention groups and each group was composed of two treatment arms: one was bevacizumab + CT, and another was CT alone. The chemotherapy regimens in these two groups were entirely different, and the analysis was performed separately; therefore, we considered it to be two independent trials, as was also the case for Sikov's study. Regimens in seven trials^9^, ^10^, ^42^, ^44^, ^47^, ^48^ were neoadjuvant chemotherapies. Study endpoints included OS, PFS, ORR, and pCR. We did not analyze DFS since only Bell's study took it as a study endpoint.^35^, ^57^ Overall, the included RCTs were of high quality, but the methodological risk of bias assessment suggested a high risk of performance might exist, which was due to the absence of a placebo (Figure 2). Therefore, we defined the treatment CT + placebo and CT as different regimens.

### 3.2 Evidence network of the comparisons and comparison‐adjusted funnel plots

**Figure 3:**
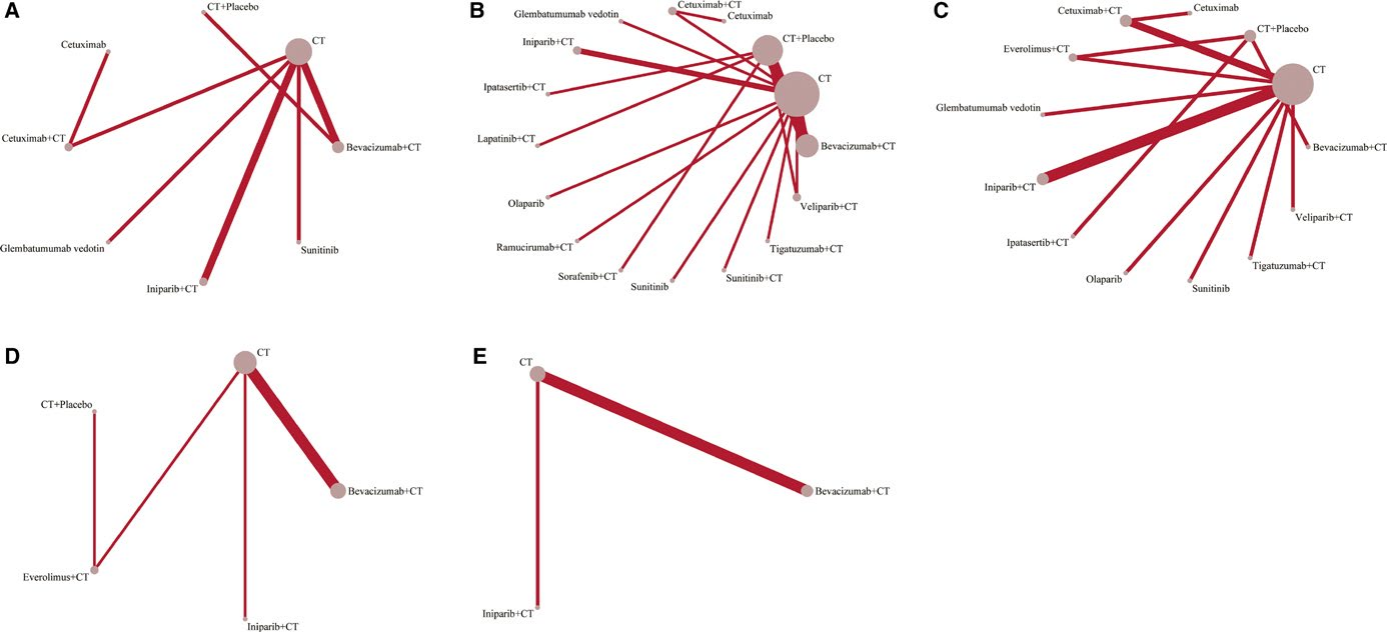
Network of the comparisons included in the network meta‐analysis. A‐E, Network of the comparisons for OS (A), PFS (B), ORR (C), pCR breast and axillary nodes (D), and pCR breast (E). Each node corresponded to a regimen included in the analysis, and each line represented direct comparisons between regimens, with node size and line width proportional to the number of trials directly comparing the regimens. (CT, chemotherapy; OS, overall survival; PFS, progression‐free survival; ORR, objective response rate; pCR, pathological complete response)

The network plots (Figure 3) displayed the comparisons of the eight treatments for OS, 16 treatments for PFS, 13 treatments for ORR, five treatments for pCR breast and axillary nodes, and three treatments for pCR breast. Bevacizumab + CT vs CT, iniparib + CT vs CT, and cetuximab + CT vs CT were the most frequent treatment regimens being directly compared. The majority of target therapies were combined with CT, yet cetuximab, glembatumumab vedotin, sunitinib, and olaparib were used as monotherapies. There were no closed triangular or quadratic loops formed by direct comparisons; consequently, loop‐specific heterogeneity estimates could not be calculated. The influence and contribution of each direct comparison to the whole network were presented in Figure S1, using weighted squares along with the respective percentages. (The contribution plot of PFS was provided in a table format thanks to the large‐scale data.).

To assess the potential small‐study effects, the comparison‐adjusted funnel plots were applied. Since there was not a single reference line against which symmetry could be judged, we made the specific assumption that target therapies alone or in combination with CT were favored in small studies. As we can see in Figure S2, symmetrical comparison‐adjusted funnel plots suggested no small‐study effects existed.

### 3.3 Bayesian network meta‐analysis

First, we evaluated the fit of the fixed or random effects model according to DIC. For OS, DIC was 6.985 in the fixed effects model, a little bit larger than 6.773 in the random effects model. However, DIC was smaller in the fixed effects model for other study endpoints, with differences of no more than 2. The Brooks‐Gelman‐Rubin diagnostic test indicated better convergence of the fixed effects model. Besides, the high level of uncertainty of the posterior standard deviation showed that there was little information to inform the random effects parameters.^24^ Therefore, we adopted the fixed effects model for network meta‐analysis.

**Figure 4:**
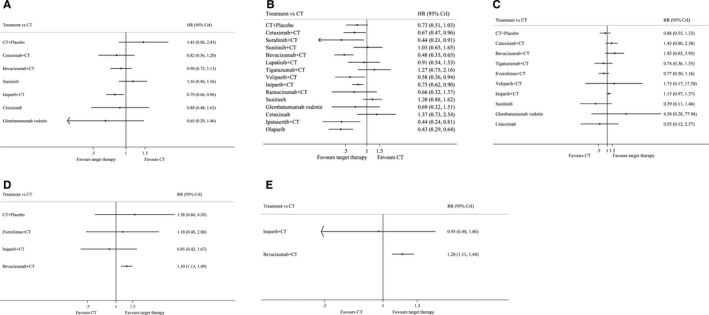
Forest plot of the estimated HR and RR for different target therapies compared with CT in the fixed effects network meta‐analysis. A and B, Forest plot of the estimated HR for different target therapies compared with CT in the fixed effects network meta‐analysis for OS (A) and PFS (B). HR and its 95% CrI <1 favored target therapies otherwise favored CT. C‐E, Forest plot of the estimated RR for different target therapies compared with CT in the fixed effects network meta‐analysis for ORR (C), pCR breast and axillary nodes (D), and pCR breast (E). RR and its 95% CrI>1 favored target therapies otherwise favored CT. (CT, chemotherapy; OS, overall survival; PFS, progression‐free survival; ORR, objective response rate; pCR, pathological complete response; CrI, credible interval)

As shown in Figure 4A and Table 2A, iniparib + CT significantly improved OS compared with CT (0.79, 0.66‐0.96) or CT + placebo (0.55, 0.31‐0.97), while bevacizumab + CT significantly improved OS compared with CT + placebo (0.62, 0.38‐1.00). In accordance with the results of PFS, we found several targeted agents were beneficial to TNBC patients. As Figure 4B and Table 2B showed, cetuximab + CT (0.67, 0.47‐0.96), sorafenib + CT (0.44, 0.21‐0.91), bevacizumab + CT (0.48, 0.35‐0.65), veliparib + CT (0.58, 0.36‐0.94), iniparib + CT (0.75, 0.62‐0.90), ipatasertib + CT (0.44, 0.24‐0.81), and olaparib (0.43, 0.29‐0.64) all significantly improved PFS compared with CT. Moreover, when in comparison with CT + placebo, bevacizumab + CT (0.66, 0.55‐0.80), ipatasertib + CT (0.60, 0.37‐0.98), and olaparib (0.59, 0.35‐0.99) showed a distinct improvement. However, it was noteworthy that iniparib + CT decreased PFS compared with bevacizumab + CT (1.55, 1.08‐2.22), suggesting that bevacizumab was superior. As for ORR, only bevacizumab + CT and olaparib showed benefits. Bevacizumab + CT significantly improved ORR compared with CT + placebo (2.15, 1.16‐4.05) and olaparib significantly improved ORR compared with CT (2.57, 1.31‐5.09; Figure 4C and Table 2C). Furthermore, bevacizumab + CT was also effective in both pCR breast and axillary nodes and pCR breast. As shown in Figure 4D,E and Table 2D‐E, bevacizumab + CT significantly improved pCR breast and axillary nodes (1.30, 1.13‐1.49) and pCR breast (1.26, 1.11‐1.44) when compared with CT.

**Table 2:** (A) Estimated HR between all treatments for OS in the fixed effects network meta‐analysis. (B) Estimated HR between all treatments for PFS in the fixed effects network meta‐analysis. (C) Estimated RR between all treatments for ORR in the fixed effects network meta‐analysis. (D) Estimated RR between all treatments for pCR breast and axillary in the fixed effects network meta‐analysis. (E) Estimated RR between all treatments for pCR breast in the fixed effects network meta‐analysis

A							
CT	1.45 (0.86‐2.45)	0.82 (0.56‐1.20)	0.90 (0.72‐1.13)	1.16 (0.86‐1.56)	0.79 (0.66‐0.96)	0.88 (0.48‐1.63)	0.65 (0.29‐1.46)
	CT + Placebo	0.57 (0.30‐1.10)	0.62 (0.38‐1.00)	0.80 (0.43‐1.48)	0.55 (0.31‐0.97)	0.61 (0.27‐1.38)	0.45 (0.17‐1.18)
		Cetuximab + CT	1.10 (0.70‐1.71)	1.42 (0.87‐2.31)	0.97 (0.64‐1.49)	1.08 (0.66‐1.74)	0.79 (0.33‐1.95)
			Bevacizumab + CT	1.29 (0.89‐1.88)	0.89 (0.66‐1.19)	0.98 (0.51‐1.90)	0.72 (0.31‐1.69)
				Sunitinib	0.69 (0.48‐0.98)	0.76 (0.38‐1.52)	0.56 (0.24‐1.34)
					Iniparib + CT	1.11 (0.58‐2.11)	0.81 (0.36‐1.90)
						Cetuximab	0.74 (0.27‐2.05)
							Glembatumumab vedotin

B															
CT	0.73 (0.51‐1.03)	0.67 (0.47‐0.96)	0.44 (0.21‐0.91)	1.03 (0.65‐1.65)	0.48 (0.35‐0.65)	0.91 (0.54‐1.53)	1.27 (0.75‐2.16)	0.58 (0.36‐0.94)	0.75 (0.62‐0.90)	0.66 (0.32‐1.37)	1.20 (0.88‐1.62)	0.69 (0.32‐1.51)	1.37 (0.73‐2.54)	0.44 (0.24‐0.81)	0.43 (0.29‐0.64)
	CT + Placebo	0.92 (0.56‐1.51)	0.60 (0.31‐1.15)	1.41 (0.80‐2.54)	0.66 (0.55‐0.80)	1.25 (0.86‐1.84)	1.74 (0.92‐3.30)	0.80 (0.51‐1.25)	1.03 (0.69‐1.53)	0.90 (0.41‐2.05)	1.65 (1.04‐2.62)	0.95 (0.40‐2.24)	1.89 (0.91‐3.83)	0.60 (0.37‐0.98)	0.59 (0.35‐0.99)
		Cetuximab + CT	0.65 (0.28‐1.47)	1.53 (0.86‐2.77)	0.72 (0.44‐1.15)	1.36 (0.72‐2.54)	1.89 (1.00‐3.61)	0.87 (0.48‐1.58)	1.11 (0.74‐1.67)	0.98 (0.45‐2.23)	1.78 (1.11‐2.85)	1.03 (0.44‐2.45)	2.04 (1.22‐3.39)	0.65 (0.32‐1.33)	0.64 (0.38‐1.09)
			Sorafenib + CT	2.36 (0.97‐5.64)	1.10 (0.56‐2.17)	2.09 (0.97‐4.44)	2.91 (1.16‐7.27)	1.34 (0.60‐2.94)	1.71 (0.78‐3.68)	1.51 (0.54‐4.25)	2.74 (1.22‐6.12)	1.58 (0.53‐4.65)	3.14 (1.16‐8.23)	1.00 (0.44‐2.27)	0.99 (0.42‐2.29)
				Sunitinib + CT	0.47 (0.27‐0.81)	0.88 (0.44‐1.76)	1.23 (0.61‐2.48)	0.57 (0.29‐1.10)	0.72 (0.44‐1.19)	0.64 (0.28‐1.51)	1.16 (0.66‐2.01)	0.67 (0.27‐1.68)	1.33 (0.60‐2.87)	0.42 (0.20‐0.91)	0.42 (0.23‐0.76)
					Bevacizumab + CT	1.89 (1.24‐2.90)	2.63 (1.42‐4.86)	1.21 (0.77‐1.91)	1.55 (1.08‐2.22)	1.36 (0.63‐3.02)	2.49 (1.61‐3.82)	1.43 (0.62‐3.34)	2.85 (1.41‐5.67)	0.90 (0.54‐1.54)	0.89 (0.55‐1.46)
						Lapatinib + CT	1.39 (0.67‐2.92)	0.64 (0.36‐1.16)	0.82 (0.47‐1.42)	0.72 (0.30‐1.79)	1.32 (0.72‐2.40)	0.76 (0.30‐1.96)	1.50 (0.65‐3.38)	0.48 (0.26‐0.90)	0.47 (0.25‐0.91)
							Tigatuzumab + CT	0.46 (0.22‐0.94)	0.59 (0.33‐1.03)	0.52 (0.21‐1.28)	0.94 (0.51‐1.74)	0.54 (0.21‐1.41)	1.08 (0.47‐2.42)	0.34 (0.15‐0.77)	0.34 (0.17‐0.66)
								Veliparib + CT	1.27 (0.76‐2.12)	1.12 (0.48‐2.69)	2.04 (1.16‐3.59)	1.18 (0.47‐2.98)	2.34 (1.05‐5.09)	0.75 (0.38‐1.46)	0.73 (0.40‐1.36)
									Iniparib + CT	0.88 (0.42‐1.88)	1.60 (1.12‐2.29)	0.92 (0.42‐2.07)	1.83 (0.95‐3.51)	0.58 (0.31‐1.11)	0.58 (0.38‐0.89)
										Ramucirumab + CT	1.81 (0.81‐3.98)	1.04 (0.35‐3.03)	2.07 (0.79‐5.38)	0.66 (0.26‐1.69)	0.65 (0.29‐1.48)
											Sunitinib	0.57 (0.25‐1.34)	1.14 (0.57‐2.29)	0.36 (0.18‐0.72)	0.36 (0.22‐0.59)
												Glembatumumab vedotin	1.98 (0.72‐5.45)	0.63 (0.23‐1.69)	0.62 (0.26‐1.51)
													Cetuximab	0.32 (0.13‐0.76)	0.31 (0.15‐0.66)
														Ipatasertib + CT	0.99 (0.48‐2.03)
															Olaparib

C												
CT	0.86 (0.55‐1.34)	1.42 (0.86‐2.36)	1.83 (0.85‐3.93)	0.74 (0.36‐1.57)	0.76 (0.50‐1.17)	1.72 (0.18‐17.57)	1.15 (0.97‐1.37)	0.40 (0.10‐1.50)	4.48 (0.27‐77.75)	0.54 (0.12‐2.48)	1.07 (0.55‐2.03)	2.57 (1.31‐5.09)
	CT + Placebo	1.66 (0.85‐3.28)	2.15 (1.16‐4.05)	0.86 (0.36‐2.04)	0.89 (0.77‐1.04)	2.02 (0.18‐21.25)	1.35 (0.83‐2.18)	0.46 (0.11‐1.87)	5.29 (0.30‐93.78)	0.63 (0.13‐3.09)	1.25 (0.77‐2.01)	2.99 (1.34‐6.85)
		Cetuximab + CT	1.29 (0.51‐3.25)	0.52 (0.21‐1.27)	0.54 (0.28‐1.04)	1.21 (0.11‐13.09)	0.81 (0.48‐1.38)	0.28 (0.07‐1.16)	3.18 (0.18‐55.95)	0.38 (0.09‐1.60)	0.75 (0.33‐1.72)	1.80 (0.78‐4.22)
			Bevacizumab + CT	0.40 (0.14‐1.17)	0.42 (0.22‐0.80)	0.94 (0.08‐10.74)	0.63 (0.28‐1.39)	0.22 (0.05‐1.01)	2.49 (0.13‐46.65)	0.29 (0.05‐1.61)	0.58 (0.27‐1.28)	1.41 (0.50‐4.02)
				Tigatuzumab + CT	1.03 (0.45‐2.41)	2.33 (0.19‐26.60)	1.56 (0.73‐3.34)	0.54 (0.12‐2.47)	6.19 (0.33‐114.40)	0.73 (0.13‐3.93)	1.45 (0.54‐3.86)	3.47 (1.27‐9.63)
					Everolimus + CT	2.26 (0.20‐23.60)	1.51 (0.95‐2.39)	0.52 (0.13‐2.10)	5.94 (0.34‐104.10)	0.71 (0.14‐3.45)	1.40 (0.85‐2.30)	3.36 (1.52‐7.52)
						Veliparib + CT	0.69 (0.07‐7.18)	0.24 (0.02‐3.55)	2.70 (0.07‐103.70)	0.32 (0.02‐5.36)	0.63 (0.06‐7.22)	1.53 (0.14‐17.69)
							Iniparib + CT	0.34 (0.09‐1.31)	3.90 (0.23‐67.76)	0.47 (0.10‐2.16)	0.92 (0.47‐1.82)	2.23 (1.10‐4.51)
								Sunitinib	11.74 (0.49‐256.70)	1.37 (0.18‐10.75)	2.74 (0.62‐12.02)	6.53 (1.47‐29.56)
									Glembatumumab vedotin	0.12 (0.01‐3.23)	0.24 (0.01‐4.53)	0.58 (0.03‐10.93)
										Cetuximab	1.99 (0.38‐10.44)	4.77 (0.89‐25.71)
											Ipatasertib + CT	2.41 (0.94‐6.24)
												Olaparib

D				
CT	1.58 (0.60‐4.20)	1.18 (0.48‐2.86)	0.85 (0.42‐1.67)	1.30 (1.13‐1.49)
	CT + Placebo	0.74 (0.50‐1.10)	0.54 (0.17‐1.80)	0.82 (0.31‐2.18)
		Everolimus + CT	0.73 (0.24‐2.25)	1.10 (0.44‐2.68)
			Iniparib + CT	1.52 (0.75‐3.09)
				Bevacizumab + CT

E		
CT	0.95 (0.48‐1.86)	1.26 (1.11‐1.44)
	Iniparib + CT	1.33 (0.66‐2.70)
		Bevacizumab + CT

Estimated HR (for OS and PFS) or RR (for ORR and pCR) and its 95% CrI between all treatments were shown in each cell. The column treatment was compared with the row treatment. A‐B. HR < 1 indicated patients in the column treatment group achieved better OS/PFS than patients in the row treatment group, and the numbers were blue if the Bayesian *P* value < 0.05. HR > 1 indicated patients in the column treatment group achieved worse OS/PFS than patients in the row group, and the numbers were red if the Bayesian *P* value < 0.05. C‐E. RR > 1 indicated patients in the column treatment group achieved better ORR/pCR than patients in the row treatment group, and the numbers were blue if the Bayesian *P* value < 0.05. RR < 1 indicated patients in the column treatment group achieved worse ORR/pCR than patients in the row group, and the numbers were red if the Bayesian *P* value < 0.05.

CI, confidence interval; CT, chemotherapy; ORR, objective response rate; OS, overall survival; pCR, pathological complete response; PFS, progression‐free survival.

The probability of rank for each treatment regimen was also calculated (Figure S3). Nevertheless, ranking of treatment regimens based merely on the probability of being the best should be avoided, since this does not account for the indeterminacy in the relative treatment effects and could illogically give higher ranks to treatment regimens for which little evidence was available.^23^ Therefore, we selected the cumulative probability rankograms (Figure S4) and calculated SUCRA, which was a plain transformation of the mean rank and accounted for both the location and the variance of the relative treatment regimen effects.^27^

**Figure 5:**
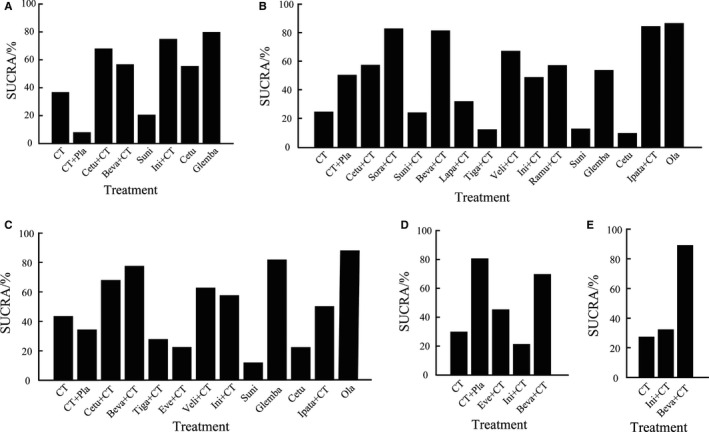
SUCRA for all treatments in the network meta‐analysis. A‐E, SUCRA histogram of different treatments for OS (A), PFS (B), ORR (C), pCR breast and axillary nodes (D), and pCR breast (E). (CT, chemotherapy; OS, overall survival; PFS, progression‐free survival; ORR, objective response rate; pCR, pathological complete response; Pla, placebo; Cetu, cetuximab; Beva, bevacizumab; Suni, sunitinib; Ini, iniparib; Glemba, glembatumumab vedotin; Sora, sorafenib; Lapa, lapatinib; Tiga, tigatuzumab; Veli, veliparib; Ramu, ramucirumab; Ipata, ipatasertib; Ola, olaparib; everolimus)

In terms of OS, glembatumumab vedotin ranked first (SUCRA: 79.8%) and iniparib + CT ranked second (74.9%), yet CT + placebo had the lowest SUCRA of 8.0% and sunitinib had the second lowest SUCRA (Figure 5A and Table S1A). As for PFS, we found olaparib, ipatasertib + CT, and sorafenib + CT were in the top three with SUCRAs of 87.6%, 85.5%, and 83.8%, respectively, yet cetuximab, tigatuzumab + CT, and sunitinib had the lowest SUCRAs, even worse than CT alone (Figure 5B and Table S1B). In terms of ORR, olaparib showed the best efficacy again with SUCRA of 87.8% and glembatumumab vedotin (82.0%) and bevacizumab + CT (77.7%) came in second and third while sunitinib had the lowest mean rank (Figure 5C and Table S1C). Yet, for pCR breast and axillary nodes, targeted therapies did not show superiority against CT + placebo, which had the highest SUCRA of 81.3% while bevacizumab + CT only ranked second (Figure 5D and Table S1D). With regard to pCR breast, bevacizumab + CT ranked first (89.5%), and iniparib + CT, and CT ranked second and third sequentially (Figure 5E and Table S1E).

### 3.4 Traditional pairwise meta‐analysis of direct comparisons

A consistency test was conducted by comparing the estimated effect sizes from the Bayesian network meta‐analysis with the pooled effect sizes from the traditional pairwise meta‐analysis. Only direct comparisons reported at least twice were considered. The fixed effects model was adopted, since no significant heterogeneity was observed according to the *I* ^2^ test. As we expected, the results of the traditional pairwise meta‐analysis were highly consistent with the results of direct comparisons in the Bayesian network meta‐analysis (Figure S5).

### 3.5 Sensitivity analysis based on the frequentist graph‐theoretical model

We also performed network meta‐analysis based on the frequentist graph‐theoretical model for sensitivity analysis.^29^ The fixed effects model was adopted, consistent with previous Bayesian network meta‐analysis. Forest plots of the efficacy of treatment regimens against CT in terms of OS, PFS, ORR, and pCR are presented in Figure S6, and the results are also highly consistent with the Bayesian network meta‐analyses in Figure 4.

Like the SUCRA value in the Bayesian network meta‐analysis, the P‐score was used for ranking treatments in the frequentist graph‐theoretical meta‐analysis.^31^ In terms of OS, glembatumumab vedotin had the highest P‐score of 0.7981 and iniparib + CT had the second highest P‐score of 0.7496, yet CT + placebo had the lowest P‐score of 0.0813 and sunitinib had the second lowest P‐score of 0.2035 (Table S2A). As for PFS, olaparib, ipatasertib + CT, and sorafenib + CT had P‐scores of 0.8764, 0.8564, and 0.8370, respectively, ranking as the top three, yet cetuximab had the lowest P‐score of 0.1083 and tigatuzumab + CT had the second lowest P‐score of 0.1309 (Table S2B). In terms of ORR, olaparib showed the highest P‐score of 0.8778 not surpirsingly, yet sunitinib had the lowest P‐score of 0.1175 (Table S2C). However, in terms of pCR breast and axillary nodes, targeted therapies or combined therapies did not show superiority against CT + placebo, and CT + placebo had the highest P‐score of 0.8130, similar to the results of SUCRA (Table S2D). As for pCR breast, bevacizumab + CT had the highest P‐score of 0.8944 with iniparib + CT and CT following behind (Table S2E). All rankings based on P‐scores in the frequentist graph‐theoretical meta‐analysis were consistent with SUCRA values in the Bayesian network meta‐analysis, which confirmed the stability of our results.

## 4 DISCUSSION

Triple‐negative breast cancer is quite a complex disease. Its relative aggressiveness and impressive heterogeneity have become challenges that clinicians face in making strides against this disease.^58^ Although several large‐scale RCTs have shown the clinical efficacies of targeted therapies, comparisons between those treatments remain uninvestigated. Therefore, we conducted a network meta‐analysis to compare the efficacy of various targeted agents in the treatment of all‐stage TNBC.

Our results suggested that the PARP inhibitor‐olaparib was the best for PFS and ORR and patients can benefit from bevacizumab + CT with respect to OS, PFS, ORR, and pCR compared with CT with or without placebo. In addition, our study also confirmed the role of some other targeted agents such as ipatasertib, cetuximab, iniparib, and sorafenib in improving survival outcomes. For example, iniparib + CT had a better effect in prolongation of OS than CT + placebo while sorafenib + CT, cetuximab + CT, and iniparib + CT acquired better PFS in comparison with CT alone. These results were quite distinct from the previous network meta‐analysis for targeted treatment of advanced TNBC.^59^ Ge et al^59^ found that only bevacizumab + CT effectively improved PFS while no significant differences in OS, PFS, and ORR were found between other agents and CT. Several distinctions existed between our studies. Ge et al focused on advanced/metastatic TNBC and categorized both CT and CT + placebo as CT, while we analyzed all strategies for all‐stage TNBC and made a distinction between CT alone and CT + placebo. Despite the placebo being considered ineffective, we found that the studies whose control group was treated with CT + placebo were double‐blinded with lower performance bias, while other studies were regarded as open‐label studies.

The rankings of treatments were made as well in our study. According to the results of treatment rank probabilities, we found that olaparib provided a best benefit over all other therapies. It is well‐known that breast cancer patients who carry germline mutations in either BRCA1 or BRCA2 are sensitive to PARP inhibitors.^60^ Although the OlympiAD trial only included patients with BRCA mutations, it was noteworthy that TNBC patients benefited more than those who were HR‐positive maybe due to the similarities in the gene‐expression profiles of BRCA1‐deficient breast cancers and sporadic TNBCs.^49^, ^55^ In addition, veliparib or iniparib + CT also showed superiority in PFS. Consequently, we consider that there is a strong rationale for treating TNBC with PARP inhibitors.

Bevacizumab is the most widely studied agent. A recent conventional meta‐analysis of additional bevacizumab as neoadjuvant therapy showed statistically significant improvements in pCR of TNBC patients,^61^ consistent with our results. Different chemotherapeutic agents all showed a greater benefit of survival outcomes or ORR when combined with chemotherapy.^37^, ^43^, ^48^, ^51^ Our results provided additional evidence that bevacizumab may be a viable treatment for TNBC. On the contrary, ramucirumab, another VEGFR‐2 antagonist, did not significantly improve the PFS of TNBC patients.^54^ It seems that antiangiogenic agents in the treatment of TNBC remain controversial.

Glembatumumab vedotin was found to have the highest SUCRA in OS, which meant it had greater probability of being effective. However, the statistical power was insufficient maybe due to the limited number of included studies and sample sizes. Glembatumumab vedotin is an ADC drug against the tumor‐associated antigen gpNMB. Previously, noteworthy activity was observed in glembatumumab vedotin‐treated TNBC patients, with a possible improvement in OS and PFS.^15^ The further subgroup analysis indicated that glembatumumab vedotin significantly improved OS and PFS for tumor gpNMB‐overexpressed TNBC patients.^15^ For those gpNMB‐overexpressed patients, glembatumumab vedotin may become a promising targeted therapy,^62^ and we expect more clinical trials carried out to confirm it.

Apart from the treatments mentioned above, immune checkpoint inhibition has received much attention in recent years. Previous studies reported that programmed cell death ligand 1 (PD‐L1) was expressed in 20% of TNBC^63^ and high PD‐L1 expression contributed to poor prognosis,^64^ suggesting the PD‐L1/PD‐1 pathway blockade as a highly promising therapy. Pembrolizumab is under study for treatment of TNBC nowadays. In the phase Ib KEYNOTE‐012 trial, pembrolizumab demonstrated an ORR of 18.5% with a median time to response of 17.9 weeks.^14^ In spite of the low response rate, a subset of patients showed long‐lasting responses,^14^ supporting further development of pembrolizumab for the treatment of TNBC.

The limitations of this study also need to be acknowledged. First of all, there were no closed loops in our network, thus, evaluation of inconsistency could not be conducted. Heterogeneity between included trials should not be ignored, since the disease stage of patients included in the present analysis was heterogenous and data did not allow us to perform subgroup analysis. Meanwhile, different study designs (double‐blinded or not) and treatment settings (neoadjuvant, adjuvant, or metastasis therapy) were both causes of heterogeneity. Second, the number of included studies was relatively small. For some agents, such as sorafenib and lapatinib, only one RCT was identified and the sample size was small. More than half of the studies were open‐label, allowing a high risk of performance bias. Maybe more blinded RCTs are needed since not entirely identical results were observed for CT + placebo and CT. Additionally, we did not distinguish the specific composition of chemotherapy across studies because different chemotherapy strategies were chosen in various trials. TNBC patients may show distinct sensitivity to different chemotherapeutic agents. For instance, a recent meta‐analysis reported significant pCR rates increase of platinum‐based neoadjuvant chemotherapy for TNBC^65^ while in our study, Baselga et al,^33^ Carey et al,^38^ and O'Shaughnessy et al^49^ used cisplatin or carboplatin as a regimen. Finally, although Bayesian network meta‐analysis is a potential solution enabling indirect comparison when a head‐to‐head trial is not available and making it realizable to rank several treatments by combining direct and indirect comparisons, it only pools the ratios rather than differences between treatments in each study. Perhaps that's why glembatumumab vedotin has great probabilities to rank first in OS and second in ORR but make no significant differences compared with CT or CT + Placebo. Although this is not the first network meta‐analysis of targeted agents for TNBC, our study may show different results, indicating that more targeted agents have potential efficacy for TNBC patients.

Nowadays, with improving understanding of biological complexity and diversity of TNBC, six different molecular subtypes were identified,^66^ which helped to develop molecular drivers that can be therapeutically targeted. In view of the limited clinical benefit with targeted agents in unselected TNBC patients of some trials, Bareche et al^67^ unraveled molecular heterogeneity of six subtypes by using an integrative genomic analysis. The researchers found specific differences in mutational and copy number profiles characterizing each subtype and offered novel therapeutic avenues for these patients.^67^ For example, basal‐like 1 (BL1) subset characterizing with high genomic instability for BRCA1/2 may be sensitive to PARP inhibitors while mesenchymal (M) subtype tumors with high level of EGFR mRNA expression may be potential candidates to anti‐VEGF inhibitors and EGFR inhibitors.^67^ Thus, the application of a comprehensive genomic characterization may help to select the most appropriate treatment in TNBC and individualized treatment for TNBC patients may be extremely necessary.^67^, ^68^ Combined with our findings, we assumed that bevacizumab may be more suitable for M subtype TNBC and olaparib, veliparib could be used in BL1 subset.

In conclusion, our network meta‐analysis suggested that addition of bevacizumab to chemotherapy could be recommended to treat TNBC patients. Olaparib had great effects in PFS and ORR especially for those with BRCA mutations. Glembatumumab vedotin ranked first in OS, without statistically significant differences compared with CT alone, but it may work well while gpNMB is overexpressed. When in combination with CT, some other targeted agents such as ipatasertib, cetuximab, sorafenib, veliparib, and iniparib showed significant superiority in PFS, exhibiting therapeutic value to some extent. Without any benefits from sunitinib, cetuximab, and tigatuzumab, we advise not to prescribe them to TNBC patients. Based on the impressive heterogeneity of TNBC, treatment personalization deserves to be promoted. Multiple targeted agents for TNBC are still at various stages of development and we expect more advances will be made in the near future.

## CONFLICT OF INTEREST

None declared.
