# Supplementary material for: A Bayesian network meta‐analysis of the efficacy of targeted therapies and chemotherapy for treatment of triple‐negative breast cancer
Source: Cancer Med. 2018 Dec 7;8(1):383–99. doi: 10.1002/cam4.1892 (PMC6346255; doi:10.1002/cam4.1892)
Supplement: Supplementary file 7 [file CAM4-8-383-s007.doc]

**Supporting Information**

**Search strategy**

The search strategy was as follows: (“breast” OR “mammary”) AND (“cancer” OR “tumor” OR “tumour” OR “carcinoma” OR “neoplasm” OR “neoplasia” OR “malignancy”) AND (“triple negative” OR “triple-negative” OR “TNBC”) AND (“methotrexate” OR “fluorouracil” OR “uracil-tegafur” OR “gemcitabine” OR “capecitabine” OR “cyclophosphamide” OR “cisplatin” OR “carboplatin” OR “oxaliplatin” OR “trabectedin” OR “doxorubicin” OR “epirubicin” OR “taxane” OR “docetaxel” OR “paclitaxel” OR “nab-paclitaxel” OR “albumin-bound paclitaxel” OR “vinorelbine” OR “eribulin” OR “ixabepilone” OR “bevacizumab” OR “ramucirumab” OR “cetuximab” OR “panitumumab” OR “seribantumab” OR “onartuzumab” OR “avelumab” OR “atezolizumab” OR “durvalumab” OR “pembrolizumab” OR “nivolumab” OR “ipilimumab” OR “tremelimumab” OR “tigatuzumab” OR “glembatumumab” OR “celecoxib” OR “iniparib” OR “olaparib” OR “veliparib” OR “rucaparib” OR “talazoparib” OR “everolimus” OR “temsirolimus” OR “rapamycin” OR “tivozanib” OR “ipatasertib” OR “tivantinib” OR “crizotinib” OR “trametinib” OR “cobimetinib” OR “vorinostat” OR “panobinostat” OR “abexinostat” OR “enzalutamide” OR “vemurafenib” OR “sonidegib” OR “palbociclib” OR “ixazomib” OR “bortezomib” OR “carfilzomib” OR “epacadostat” OR “gefitinib” OR “erlotinib” OR “icotinib” OR “sorafenib” OR “sunitinib” OR “pazopanib” OR “vandetanib” OR “lapatinib” OR “apatinib” OR “afatinib” OR “dasatinib” OR “crizotinib” OR “axitinib” OR “cabozantinib” OR “chemotherapy” OR “adjuvant chemotherapy” OR “neoadjuvant chemotherapy” OR “target therapy”). Two independent investigators (Huihui Chen and Wei Lu) were involved in the literature search and carefully scrutinized the literatures from the initial search.

**Supplementary tables**

| Table S1A. SUCRA, PrBest and mean rank for all treatments for OS in the network meta-analysis | | | | |
| --- | --- | --- | --- | --- |
| Treatment | Fixed effects model | | |  |
|  | SUCRA/% | PrBest | Mean rank |  |
| CT | 36.8 | 0.0 | 5.4 |  |
| CT+Placebo | 8.0 | 0.2 | 7.4 |  |
| Cetuximab+CT | 67.9 | 11.2 | 3.2 |  |
| Bevacizumab+CT | 56.7 | 3.1 | 4.0 |  |
| Sunitinib | 20.5 | 0.1 | 6.6 |  |
| Iniparib+CT | 74.9 | 13.8 | 2.8 |  |
| Cetuximab | 55.4 | 14.2 | 4.1 |  |
| Glembatumumab vedotin | 79.8 | 57.4 | 2.4 |  |

| Table S1B. SUCRA, PrBest and mean rank for all treatments for PFS in the network meta-analysis | | | |
| --- | --- | --- | --- |
| Treatment | Fixed effects model | | |
|  | SUCRA/% | PrBest | Mean rank |
| CT | 25.5 | 0.0 | 12.2 |
| CT+Placebo | 51.3 | 0.0 | 8.3 |
| Cetuximab+CT | 58.3 | 0.3 | 7.3 |
| Sorafenib+CT | 83.8 | 30.5 | 3.4 |
| Sunitinib+CT | 25.0 | 0.0 | 12.2 |
| Bevacizumab+CT | 82.4 | 3.5 | 3.6 |
| Lapatinib+CT | 32.8 | 0.0 | 11.1 |
| Tigatuzumab+CT | 13.1 | 0.0 | 14.0 |
| Veliparib+CT | 68.1 | 2.4 | 5.8 |
| Iniparib+CT | 49.7 | 0.0 | 8.5 |
| Ramucirumab+CT | 58.1 | 5.6 | 7.3 |
| Sunitinib | 13.6 | 0.0 | 14.0 |
| Glembatumumab vedotin | 54.7 | 5.5 | 7.8 |
| Cetuximab | 10.6 | 0.0 | 14.4 |
| Ipatasertib+CT | 85.5 | 26.2 | 3.2 |
| Olaparib | 87.6 | 26.0 | 2.9 |

| Table S1C. SUCRA, PrBest and mean rank for all treatments for ORR in the network meta-analysis | | | |
| --- | --- | --- | --- |
| Treatment | Fixed effects model | | |
|  | SUCRA% | PrBest | Mean rank |
| CT | 43.6 | 0.0 | 7.8 |
| CT+Placebo | 34.5 | 0.0 | 8.9 |
| Cetuximab+CT | 68.1 | 0.7 | 4.8 |
| Bevacizumab+CT | 77.7 | 5.8 | 3.7 |
| Tigatuzumab+CT | 28.0 | 0.0 | 9.6 |
| Everolimus+CT | 22.6 | 0.0 | 10.3 |
| Veliparib+CT | 62.9 | 19.2 | 5.4 |
| Iniparib+CT | 57.8 | 0.0 | 6.1 |
| Sunitinib | 12.0 | 0.1 | 11.6 |
| Glembatumumab vedotin | 82.0 | 55.3 | 3.2 |
| Cetuximab | 22.5 | 0.6 | 10.3 |
| Ipatasertib+CT | 50.3 | 0.1 | 7.0 |
| Olaparib | 87.8 | 18.1 | 2.5 |

| Table S1D. SUCRA, PrBest and mean rank for all treatments for pCR breast and axillary in the network meta-analysis | | | |
| --- | --- | --- | --- |
| Treatment | Fixed effects model | | |
|  | SUCRA/% | PrBest | Mean rank |
| CT | 30.5 | 0.0 | 3.8 |
| CT+Placebo | 81.3 | 61.3 | 1.7 |
| Everolimus+CT | 45.9 | 2.6 | 3.2 |
| Iniparib+CT | 21.9 | 5.7 | 4.1 |
| Bevacizumab+CT | 70.4 | 30.4 | 2.2 |

| Table S1E. SUCRA, PrBest and mean rank for all treatments for pCR breast in the network meta-analysis | | | |
| --- | --- | --- | --- |
| Treatment | Fixed effects model | | |
|  | SUCRA/% | PrBest | Mean rank |
| CT | 27.8 | 0.0 | 2.4 |
| Iniparib+CT | 32.7 | 21.0 | 2.3 |
| Bevacizumab+CT | 89.5 | 79.0 | 1.2 |

SUCRA, PrBest and mean rank for all treatments for OS(A), PFS(B), ORR(C), pCR breast and axillary nodes(D) and pCR breast(E) were shown, and larger SUCRA value indicated patients achieved better outcome in the treatment group. (CT: Chemotherapy; OS: Overall survival; PFS: Progression free survival; ORR: Objective response rate; pCR: Pathological complete response; SUCRA: Surface under the cumulative ranking curve; PrBest: Probability for each treatment of being the best)

| Table S2A. P-score for OS in the network meta-analysis based on the frequentist graph-theoretic model | | |
| --- | --- | --- |
| Treatment | P-score | Rank |
| CT | 0.3683 | 6 |
| CT+Placebo | 0.0813 | 8 |
| Cetuximab+CT | 0.6778 | 3 |
| Bevacizumab+CT | 0.5663 | 4 |
| Sunitinib | 0.2035 | 7 |
| Iniparib+CT | 0.7496 | 2 |
| Cetuximab | 0.5551 | 5 |
| Glembatumumab vedotin | 0.7981 | 1 |

| Table S2B. P-score for PFS in the network meta-analysis based on the frequentist graph-theoretic model | | | |  |
| --- | --- | --- | --- | --- |
| Treatment | P-scores | Rank | |  |
| CT | 0.2552 | 12 | |  |
| CT+Placebo | 0.5121 | 9 | |  |
| Cetuximab+CT | 0.5847 | 6 | |  |
| Sorafenib+CT | 0.8370 | 3 | |  |
| Sunitinib+CT | 0.1346 | 13 | |  |
| Bevacizumab+CT | 0.8226 | 4 | |  |
| Lapatinib+CT | 0.3293 | 11 | |  |
| Tigatuzumab+CT | 0.1309 | 15 | |  |
| Veliparib+CT | 0.6790 | | 5 | |
| Iniparib+CT | 0.4966 | | 10 | |
| Ramucirumab+CT | 0.5803 | | 7 | |
| Sunitinib | 0.1346 | | 14 | |
| Glembatumumab vedotin | 0.5473 | | 8 | |
| Cetuximab | 0.1083 | | 16 | |
| Ipatasertib+CT | 0.8564 | | 2 | |
| Olaparib | 0.8764 | | 1 | |

| Table S2C. P-score for ORR in the network meta-analysis based on the frequentist graph-theoretic model | | | |  |
| --- | --- | --- | --- | --- |
| Treatment | P-score | Rank | |  |
| CT | 0.4359 | 8 | |  |
| CT+Placebo | 0.3461 | 9 | |  |
| Cetuximab+CT | 0.6807 | 4 | |  |
| Bevacizumab+CT | 0.7760 | 3 | |  |
| Tigatuzumab+CT | 0.2785 | 10 | |  |
| Everolimus+CT | 0.2263 | 11 | |  |
| Veliparib+CT | 0.6281 | 5 | |  |
| Iniparib+CT | 0.5773 | 6 | |  |
| Sunitinib | 0.1175 | | 13 | |
| Glembatumumab vedotin | 0.8237 | | 2 | |
| Cetuximab | 0.2263 | | 11 | |
| Ipatasertib+CT | 0.5057 | | 7 | |
| Olaparib | 0.8778 | | 1 | |

| Table S2D. P-score for pCR in the network meta-analysis based on the frequentist graph-theoretic model | | |
| --- | --- | --- |
| Treatment | P-score | Rank |
| CT | 0.3040 | 4 |
| CT+Placebo | 0.8130 | 1 |
| Everolimus+CT | 0.4588 | 3 |
| Iniparib+CT | 0.2226 | 5 |
| Bevacizumab+CT | 0.7016 | 2 |

| Table S2E. P-score for pCR breast in the network meta-analysis based on the frequentist graph-theoretic model | | |
| --- | --- | --- |
| Treatment | P-score | Rank |
| CT | 0.2794 | 3 |
| Iniparib+CT | 0.3262 | 2 |
| Bevacizumab+CT | 0.8944 | 1 |

P-scores for OS(A),PFS(B), ORR(C), pCR breast and axillary nodes(D) and pCR breast(E) based on the point estimates and standard errors of the frequentist graph-theoretic model were shown, and larger P-score indicated patients achieved better outcome in the treatment group. (CT: Chemotherapy; OS: Overall survival; PFS: Progression free survival; ORR: Objective response rate; pCR: Pathological complete response)

**Supplementary figure legends**

**Figure Legends**

**Figure S1. Contribution plot of the comparisons included in the network meta-analysis.**

A-E. Contribution plot of the comparisons for OS(A), PFS(B), ORR(C), pCR breast and axillary nodes(D) and pCR breast(E). Those direct comparisons were on the horizontal axis and those network summary comparisons including both direct and indirect comparisons were on the vertical axis. The size of each square was proportional to the weight attached to each direct comparison for the estimation of each network summary effect, with the number on each square quantified the weight as percentage. The contribution of each direct comparison to the total network was shown in the “Entire network” row. (CT: Chemotherapy; OS: Overall survival; PFS: Progression free survival; ORR: Objective response rate; pCR: Pathological complete response)

**Figure S2. Comparison-adjusted funnel plot of the comparisons included in the network meta-analysis.**

A-E. Comparison-adjusted funnel plot of the comparisons for OS(A), PFS(B), ORR(C), pCR breast and axillary nodes(D) and pCR breast(E). The vertical red line represented the null hypothesis that the study specific effect sizes did not differ from the respective comparison-specific pooled effect estimates, and the line crossed the vertical red line was the regression line. Different comparisons were shown in different colors. (CT: Chemotherapy; OS: Overall survival; PFS: Progression free survival; ORR: Objective response rate; pCR: Pathological complete response)

**Figure S3. Probability rankograms for all treatments in the fixed effects network meta-analysis.**

The x-axis represented ranking and the y-axis represented probability. A-E. Probability rankogram for OS(A), PFS(B), ORR(C), pCR breast and axillary nodes(D) and pCR breast(E) in the fixed effects network meta-analysis. Higher ranking indicated better OS, PFS, ORR or pCR. (CT: Chemotherapy; OS: Overall survival; PFS: Progression free survival; ORR: Objective response rate; pCR: Pathological complete response)

**Figure S4. Cumulative probability rankograms for all treatments in the fixed effects network meta-analysis.**

The x-axis represented ranking and the y-axis represented cumulative probability. (A) Cumulative probability rankogram for OS(A), PFS(B), ORR(C), pCR breast and axillary nodes(D) and pCR breast(E) in the fixed effects network meta-analysis. Higher ranking indicated better OS, PFS, ORR or pCR. (CT: Chemotherapy; OS: Overall survival; PFS: Progression free survival; ORR: Objective response rate; pCR: Pathological complete response)

**Figure S5. Forest plot of traditional pairwise meta-analysis of direct comparisons**

A. Forest plot of the estimated HR for bevacizumab + CT compared with CT in the pairwise meta-analysis for OS. HR and its 95% CI < 1 favored bevacizumab + CT otherwise favored CT. B. Forest plot of the estimated HR for iniparib +CT compared with CT in the pairwise meta-analysis for OS. HR and its 95% CI < 1 favored iniparib +CT otherwise favored CT. C. Forest plot of the estimated HR for bevacizumab + CT compared with CT in the pairwise meta-analysis for PFS. HR and its 95% CI < 1 favored bevacizumab + CT otherwise favored CT. D. Forest plot of the estimated HR for bevacizumab + CT compared with CT + placebo in the pairwise meta-analysis for PFS. HR and its 95% CI < 1 favored bevacizumab + CT otherwise favored CT + placebo. E. Forest plot of the estimated HR for iniparib +CT compared with CT in the pairwise meta-analysis for PFS. HR and its 95% CI < 1 favored iniparib +CT otherwise favored CT. F. Forest plot of the estimated RR for cetuximab +CT compared with CT in the pairwise meta-analysis for ORR. RR and its 95% CI > 1 favored cetuximab +CT otherwise favored CT. G. Forest plot of the estimated RR for iniparib +CT compared with CT in the pairwise meta-analysis for ORR. RR and its 95% CI > 1 favored iniparib +CT otherwise favored CT. H. Forest plot of the estimated RR for bevacizumab + CT compared with CT in the pairwise meta-analysis for pCR breast and axillary nodes. RR and its 95% CI > 1 favored bevacizumab + CT otherwise favored CT. I. Forest plot of the estimated RR for bevacizumab + CT compared with CT in the pairwise meta-analysis for pCR breast. RR and its 95% CI > 1 favored Bevacizumab + CT otherwise favored CT.

**Figure S6. Forest plot of the estimated HR and RR for different target therapies compared with CT in the network meta-analysis based on the frequentist graph-theoretic model**

A&B. Forest plot of the estimated HR for different target therapies compared with CT in the fixed effects network meta-analysis based on the frequentist graph-theoretic model for OS(A) and PFS(B). HR and its 95% CI < 1 favored target therapies otherwise favored CT. C-E. Forest plot of the estimated RR for different target therapies compared with CT in the fixed effects network meta-analysis based on the frequentist graph-theoretic model for ORR(C), pCR breast and axillary nodes(D) and pCR breast(E). RR and its 95% CI > 1 favored target therapies otherwise favored CT. (CT: Chemotherapy; OS: Overall survival; PFS: Progression free survival; ORR: Objective response rate; pCR: Pathological complete response; CI: Confidence interval)
